# NodD1 and NodD2 Are Not Required for the Symbiotic Interaction of *Bradyrhizobium* ORS285 with Nod-Factor-Independent *Aeschynomene* Legumes

**DOI:** 10.1371/journal.pone.0157888

**Published:** 2016-06-17

**Authors:** Nico Nouwen, Joel Fardoux, Eric Giraud

**Affiliations:** IRD, Laboratoire des Symbioses Tropicales et Méditerranéennes, UMR IRD/ SupAgro/INRA/ UM2 /CIRAD, Montpellier, France; Estacion Experimental del Zaidin - CSIC, SPAIN

## Abstract

Photosynthetic *Bradyrhizobium* strain ORS285 forms nitrogen-fixing nodules on the roots and stems of tropical aquatic legumes of the *Aeschynomene* genus. Depending on the *Aeschynomene* species, this symbiotic interaction does or does not rely on the synthesis of Nod-factors (NFs). However, whether during the interaction of *Bradyrhizobium* ORS285 with NF-independent *Aeschynomene* species the *nod* genes are expressed and if the general regulator NodD plays a symbiotic role is unknown. Expression studies showed that in contrast to the interaction with the NF-dependent *Aeschynomene* species, *A*. *afraspera*, the *Bradyrhizobium* ORS285 *nod* genes are not induced upon contact with the NF-independent host plant *A*. *indica*. Mutational analysis of the two *nodD* genes present in ORS285, showed that deletion of *nod*D1 and *nod*D2 did not affect the symbiotic interaction between *Bradyrhizobium* ORS285 and *A*. *indica* whereas the deletions had an effect on the symbiotic interaction with *A*. *afraspera* plants. In addition, when the expression of *nod* genes was artificially induced by adding naringenin to the plant growth medium, the nodulation of *A*. *indica* by *Bradyrhizobium* ORS285 is delayed and resulted in lower nodule numbers.

## Introduction

Legume plants have developed symbiotic associations with specific soil bacteria, collectively referred to as rhizobia, which allow the plants to grow in nitrogen poor soils. This symbiosis results in the formation of a new plant organ, the nodule, cells that are intracellularly infected by the rhizobia which differentiate into nitrogen-fixing bacteroids. Some *Aeschynomene* spp. belonging to the Dalbergioid clade, a pantropical large group of papilionoid legumes, symbiotically interact with photosynthetic bradyrhizobia [1,2] and have the unusual property of forming nodules on both the root and the stem [3]. This stem nodulation can be very profuse resulting in high nitrogen fixation rates. This particularity renders some *Aeschynomene* spp. ideal candidate as “green manure” for rice production [4]. Among the photosynthetic bradyrhizobia two groups of host specificity can be distinguished [5]. Group I contains strains with a broad host range that extends to all stem nodulating *Aeschynomene species*, whereas group II contains strains that are only able to nodulate a few stem nodulating *Aeschynomene* species, including *A*. *indica*, *A*. *evenia* and *A*. *sensitiva*. Sequencing the genomes of two photosynthethic bradyrhizobia belonging to host specificity group II, i.e. ORS278 and BTAi1, showed that the genes coding for enzymes involved in synthesis of the core structure of Nod-factors (NFs; *nod*ABC) were absent [6]. This indicates that some photosynthetic *Bradyrhizobium* spp. use a novel mechanism, independent of the long-time considered universal NFs, to symbiotically interact with *Aeschynomene* plants.

The absence of *nod* genes is not a general rule among photosynthetic bradyrhizobia. Group I strains, such as the model strain *Bradyrhizobium* ORS285, do contain *nodABC* genes [7]. Interestingly, these *nod* genes are essential for the symbiosis with some *Aeschynomene* spp., such as *A*. *afraspera*, but they are dispensable for symbiosis with *Aeschynomene* spp. nodulated by group II strains, such as *A*. *indica* [6]. Thus, two types of interactions can be considered in *Aeschynomene*–photosynthetic *Bradyrhizobium* symbiosis: 1) a classical one which is NF-dependent and 2) an atypical one which is NF-independent and it is the *Aeschynomene* species that determines the *modus operandi*.

In the classical NF-dependent symbiosis, initiation of nodule development involves the exchange of molecular signals between both partners. Flavonoids exuded by plant roots are supposed to be the first signals received by the rhizobia. Flavonoids likely diffuse into the rhizobial cytoplasm where they interact with NodD proteins that belong to the LysR family of transcriptional regulators, and trigger a signal transduction cascade that plays a role in the infection process [8]. Studies of genomes of rhizobia indicate that, depending on the rhizobial species, there are one to five copies of *nodD*. In species that possess only one *nod*D copy a mutation usually results in the loss of nodulation, whereas, in the presence of multiple copies, mutations do not always affect nodulation [9–11]. In the genome of *Bradyrhizobium* ORS285 two genes have been annotated as *nod*D (*nod*D1 and *nod*D2)[12]. NodD proteins have been shown to binds to conserved 49 bp motifs (so called nod-boxes) that are found in the promoters of nodulation (*nod*, *nol* and *noe*) genes [13,14]. Most nodulation genes that are expressed in a flavonoid- and NodD-dependent manner are involved in the synthesis and secretion of NFs, rhizobial lipochito-oligosaccharide signal molecules (LCOs). NFs are recognized by plant kinases of the LysM-RLKs family, which, upon activation, initiate a developmental program in the legume host leading to the formation of the nodule structure [15]. Besides induction of genes involved in the synthesis of NFs, flavonoid- and NodD-dependent expression has been demonstrated for genes involved in the synthesis of hopanoids, rhizopine catabolism, nitrogen fixation, synthesis and/or modification of polysaccharides and expression of transcriptional regulators (TtsI) involved in the expression of Type III secretion systems [16]. As all these processes have been suggested to play a role in the establishment of a successful nodulation, the NodD protein(s) can be designed as the central regulator(s) in rhizobial nodulation.

Upon naringenin addition to the growth medium, the major NF synthesized by *Bradyrhizobium* strain ORS285 is a pentameric lipochitooligosaccharide (LCO) with a 2-O-methylfucose at the reducing end [12]. This suggests that the interaction between ORS285 strain and NF-dependent *Aeschynomene* species involves a classical molecular dialogue (i.e. induction by host plant flavonoids and subsequent synthesis of NF). However, whether the ORS285 strain uses the same or a different molecular dialogue when interacting with NF-independent *Aeschynomene* plants is not known. Therefore, the aim of this study was to determine whether the *nod*-genes of the ORS285 strain are expressed during the interaction with NF-independent *Aeschynomene* plants, and to determine the role of the nodulation regulators NodD1 and NodD2 in this interaction.

## Materials and Methods

### Bacterial strains and growth conditions

*Bradyrhizobium* ORS285 [5] and derivatives, were grown in modified YM medium [17] or a minimal growth medium (BNM-B; pH 6.8) at 37°C. Tables (S1 and S2 Tables) and a detailed description of the construction of strains and plasmids used in this study (S1 File) are given in the Supporting information. BNM-B is a synthetic plant growth medium (Buffered Nodulation Medium; [18]) that has been supplemented with succinate (10 mM), glutamate (6 mM) and a cocktail of vitamins (0.2 μg/ml riboflavin, 0.12 μg/ml biotin, 0.8 μg/ml thiamine-HCl, 0.5 μg/ml myo-inositol, 0.1 μg/ml p-aminobenzoic acid, 0.5 μg/ml nicotinic acid, 0.8 μg/ml calcium pantohenate, 1 ng/ml cyanocobalamin) to support growth of *Bradyrhizobium* strains.

### Root exudate isolation and qualitative analysis by high performance liquid chromatography

*A*. *afraspera* and *A*. *indica* seeds were surface sterilized and germinated on 0.8% agar plates. *After germination*, seedlings were grown for 7 days in 48-well plates containing 5.8 ml BNM medium [19]. Root exudate of eight 48-well plates (total volume: ~ 2 liter) was filtered (45 μm filter) and concentrated to ~100 ml using a rota evaporator (40°C). The concentrated root exudate was centrifuged (15 minutes *g) and flavonoids present in the exudate were isolated by solid phase extraction using a SepPak C18 column (1 column / 25 ml concentrated root exudate). The eluted fractions (in 100% methanol) were combined, dried under vacuum and the weight of the dry pellet was determined. The flavonoids were dissolved in 50% ethanol (~20 mg/ml) and 150 μg of material was analysed on a high-performance liquid chromatography system (Waters 2690 separation module, Waters, Milford, Massachusetts, USA) equipped with a analytic C18 reverse phase column (5 μm, 4.6 mm x 250 mm, Symmetry C18, Waters) using an isocratic gradient of solvent A (water-methanol, 70:30 [vol/vol]) for 5 min, followed by a sixty-minutes linear gradient from solvent A to solvent B (100% methanol) and finally an isocratic gradient of solvent B for 10 min, at a flow rate of 1 ml min-1. The eluent of the HPLC column was monitored at 206–400 nm.

### *Nod* gene inducing capabilities of pure flavonoids and root exudate

The *Bradyrhizobium* ORS285 *nod*A-*lac*Z reporter strain was used to assess the capacities of root exudates from *A*. *indica* and *A*. *afraspera*, respectively, to induce the expression of the *nod*A-*lac*Z transcriptional fusion. Purified flavonoids were purchased from Extrasynthese (Genay, France) or Sigma-Aldrich (St. Louis, USA). *Bradyrhizobium* ORS285::*nod*A-*lac*Z was grown in BNM-B medium till an optical density of OD600 ~ 0.4. Subsequently, the bacterial culture was diluted into fresh BNM-B

medium (OD600 = 0.1) and supplemented with root exudate or pure flavonoids. After 24 hours of growth at 28°C, the absorbance at 600 nm of the cultures were determined and β-galactosidase activities were measured according to the method of Miller (Miller 1972). Experiments were at least repeated twice with three technical replicates in one experiment.

### Plant growth and acetylene reduction assay

Sterilization of seeds, germination, plant growth and inoculation with bacterial strains were as described [12]. At 4, 5, 6, 7, 8, 10, 12 and 14 days after inoculation, the number of nodules on the roots were counted. The acetylene reduction assay (ARA) was used to measure the nitrogenase enzyme activity 14 days after inoculation [12].

## Results

### Root exudate of a NF-independent *Aeschynomene* species does not induce *nod* gene expression in *Bradyrhizobium* ORS285

**Fig 1:**
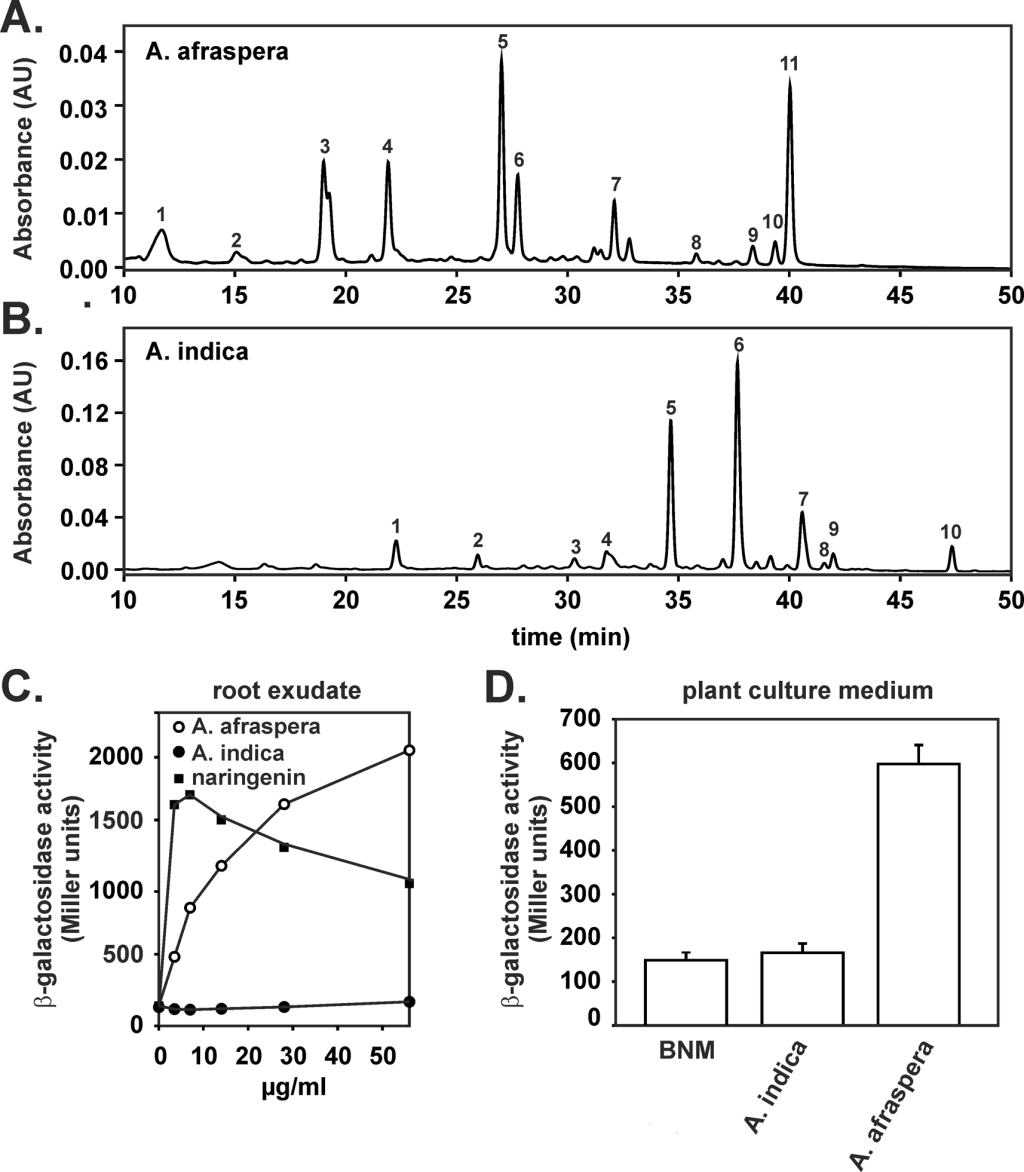
*Bradyrhizobium* ORS285 *nod* genes are not expressed upon contact with NF-independent *Aeschynomene* plants. (A) chromatogram of SepPak C18 bound material of root exudate from *A*. *afraspera* (absorbance measured at 295 nm). (B) chromatogram of SepPak C18 bound material of root exudate from *A*. *indica* (absorbance measured at 295 nm). The UV-VIS spectra of the numbered peaks as indicated in (A) and (B) can be found in the Supporting information (S1 and S2 Figs). (C) dose dependence of root exudate / naringenin-induced β-galactosidase activity of the *nod*A-*lac*Z fusion in *Bradyrhizobium* ORS285 and (D) β-galactosidase activity of *Bradyrhizobium* ORS285::*nod*A-*lac*Z cells grown for three days in the presence of *A*. *afraspera* and *A*. *indica* plants, respectively. The results are from one representative experiment with three technical replicates for each experimental condition.

**Fig 2:**
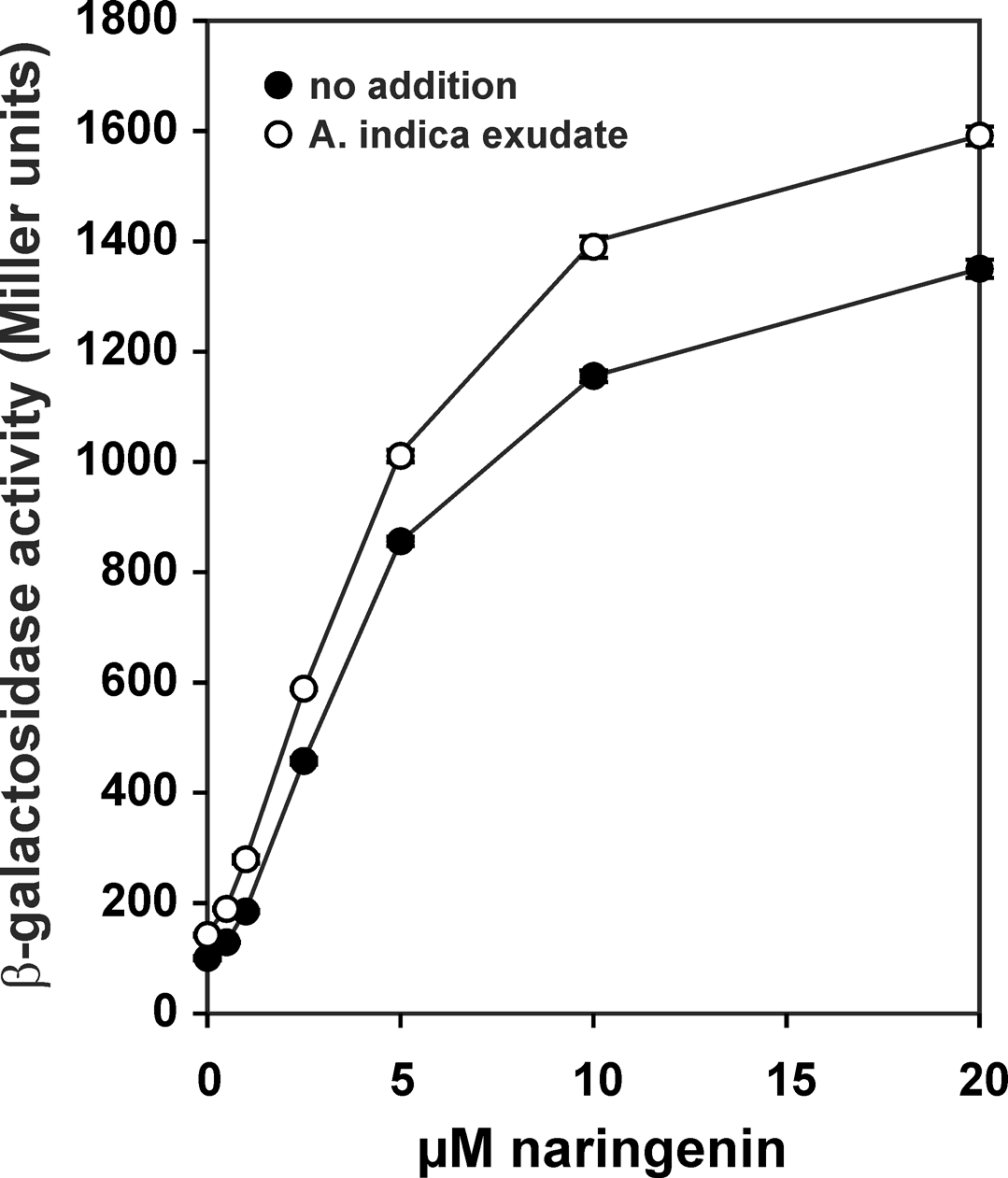
*A*. *indica* root exudate does not inhibit the naringenin induced expression of the *nod*A-*lac*Z fusion in *Bradyrhizobium* ORS285. *Bradyrhizobium* ORS285::*nod*A-*lac*Z cells were grown for 24 hr at 28°C with increasing amounts of naringenin in the absence or presence of 25 μg / ml (final concentration) SepPak C18 extracted root exudate of *A*.*indica* whereafter the β-galactosidase activity was measured. The results are from one representative experiment with three technical replicates for each experimental condition.

*Bradyrhizobium* ORS285 interacts with *A*. *afraspera* and *A*. *indica* using a NF-dependent and NF-independent process, respectively. To determine whether *nod* genes are induced upon interaction with NF-independent *Aeschynomene* plants, we extracted and concentrated the root exudate of 7-day old *A*. *indica* seedlings using a C18 solid-phase extraction protocol. As control, we also extracted root exudate of the NF-dependent host plant, *A*. *afraspera*. Qualitative analysis of the C18-extracted root exudate by reversed phase high-performance liquid chromatography showed clear differences in chromatographic pattern between the two *Aeschynomene* species (Fig 1A and 1B). The UV-VIS spectra of the majority of peaks present in the chromatograms of both root exudates contained two absorbance bands (one in the 310–385 nm region, band A and one in the 250–295 nm region, band B) which are typical for flavonoids (S1 and S2 Figs). To determine the expression of the *nod* genes (*nod*A-J operon) upon root exudate addition, we used a *Bradyrhizobium* ORS285 reporter strain which contained a *nod*A-*lac*Z transcriptional fusion on the chromosome. In the absence or presence of root exudate from *A*. *indica* plants, we measured a low (basal) β-glactosidase activity with the reporter strain (Fig 1C). In contrast, upon addition of the flavonoid naringenin or the root exudate of *A*. *afraspera* plants to the bacterial growth medium, we measured a concentration-dependent increase in the β-galactosidase activity (Fig 1C). During the solid phase extraction procedure, flavonoids or other molecules that induce *nod* gene expression could have been lost. To analyze this possibility, we inoculated 7-day old seedlings with the reporter strain and measured the β-galactosidase activity of the cells present in the plant culture medium after 3 days of incubation. Cells present in the plant culture medium of *A*. *afraspera* plants have a 3-fold higher β-galactosidase activity as compared to cells present in plant culture medium of *A*. *indica* plants or cells that had been incubated in plant growth medium alone (Fig 1D). To analyze whether *A*. *indica* root exudate contains compounds that inhibit *nod*-gene expression, we grew the ORS285 *nod*A-*lac*Z reporter strain with a high concentration of C18-extracted *A*. *indica* exudate and increasing amounts of naringenin. In the presence of *A*. *indica* root exudate, the naringenin-dependent increase in β-galactosidase -activity was under all conditions slightly higher as observed with cells grown in the presence of naringenin alone (Fig 2). Based on above results, we concluded that *A*. *indica* root exudate does not contain molecules that induce or repress *nod*-gene-expression in *Bradyrhizobium* ORS285 cells.

### NodD1 and NodD2 are not required for NF-independent nodulation of *A*. *indica* plants

**Fig 3:**
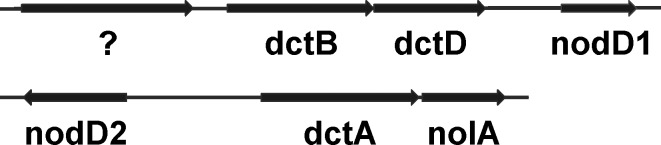
Schematic representation of the genomic regions containing the *nod*D1 and *nod*D2 gene of *Bradyrhizobium* ORS285, respectively.

**Fig 4:**
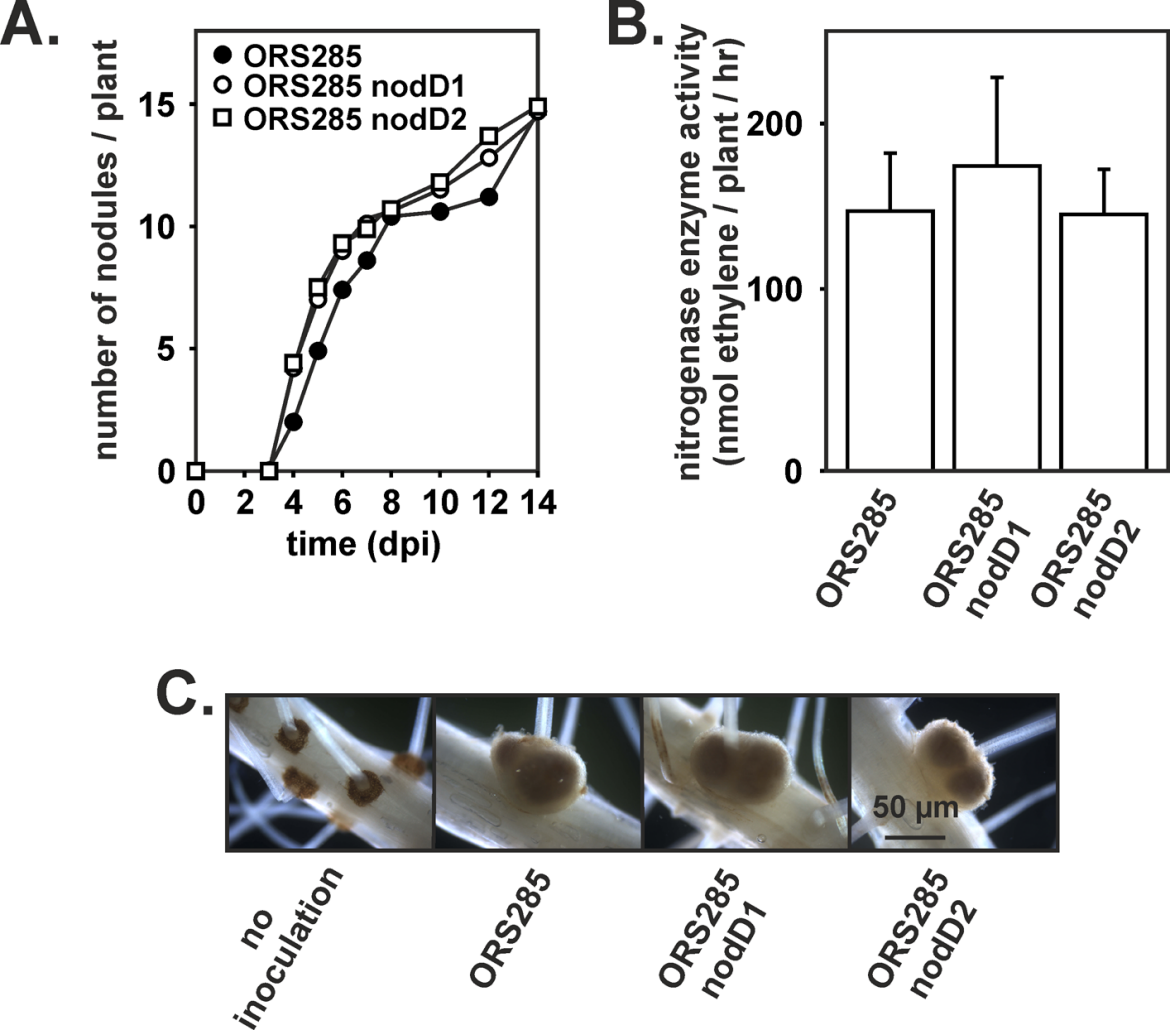
Nodulation kinetics of *Bradyrhizobium* ORS285 and Δ*nod*D1 and Δ*nod*D2 derivatives on *A*. *indica* plants. (A) The average number of nodules per plant (n = 10) at various days post infection (dpi) is presented. (B) Acetylene reducing activity in *A*. *indica* plants inoculated with *Bradyrhizobium* ORS285 and Δ*nod*D1 and Δ*nod*D2 derivatives at 14 dpi. The average amount of produced ethylene per hour and per plant is indicated. Error bars represent standard deviations (n = 10). (C) Mature nodules of *A*. *indica* plants inoculated with *Bradyrhizobium* ORS285 and Δ*nod*D1 and Δ*nod*D2 derivatives at 14 dpi.

**Fig 5:**
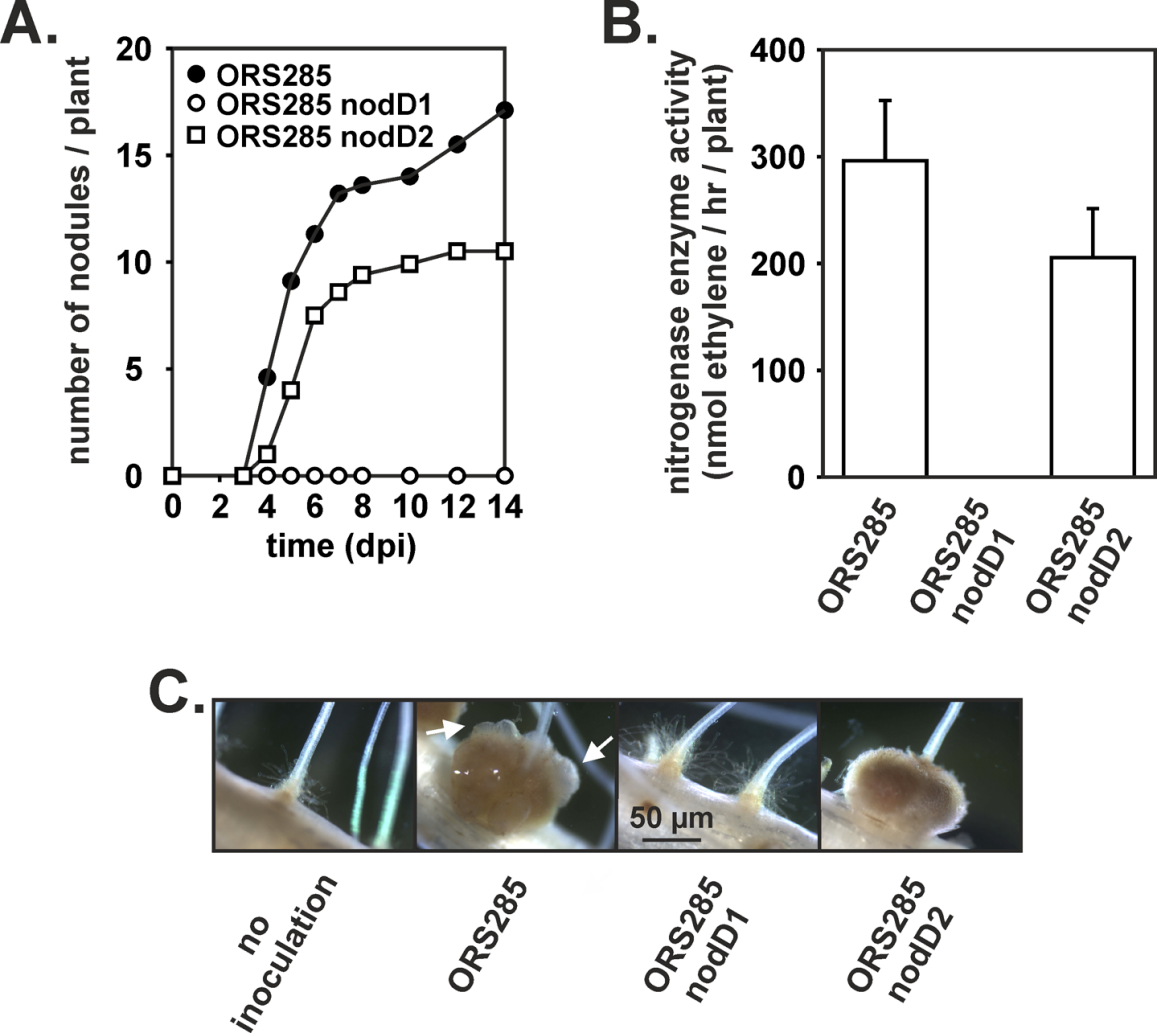
Nodulation kinetics of *Bradyrhizobium* ORS285 and Δ*nod*D1 and Δ*nod*D2 derivatives on *A*. *afraspera* plants. (A) The average number of nodules per plant (n = 10) at various days post infection (dpi) is presented. (B) Acetylene reducing activity in *A*. *afraspera* plants inoculated with *Bradyrhizobium* ORS285 and Δ*nod*D1 and Δ*nod*D2 derivatives at 14 dpi. The average amount of produced ethylene per hour and per plant is indicated. Error bars represent standard deviations (n = 10). (C) Mature nodules of *A*. *afraspera* plants inoculated with *Bradyrhizobium* ORS285 and Δ*nod*D1 and Δ*nod*D2 derivatives at 14 dpi. White arrows indicate the superficial outgrowth on nodules.

Like *B*. *diazoefficiens* USDA110, *Bradyrhizobium* ORS285 contains two genes (*nodD1* and *nodD2;* accession numbers: FQ790406 and FQ790405, respectively) encoding for the symbiotic regulator NodD [12]. Both *nod*D genes are flanked by genes involved in C4 dicarboxylate metabolism (*nod*D1, *dct*B/D; *nod*D2, *dct*A). In addition, *nod*D2 is found close to a putative *nol*A gene which encodes a protein involved in the cell density control mechanism of NF biosynthesis in *B*. *diazoefficiens* [20], (Fig 3). The *nod*D1 and *nod*D2 genes were inactivated by deletion of more than 95% of the coding region and insertion of a chloramphenicol and streptomycine resistance cassette (omega interposon) in the remaining part of the gene, respectively. *Bradyrhizobium* strain ORS285 forms nodules on *A*. *indica* using a NF-independent symbiotic process [6]. To investigate whether *nod*D1 or *nod*D2 deletion affects the symbiosis with *A*. *indica*, we infected this plant with the ORS285 Δ*nodD1* and ORS285 Δ*nodD2* mutant, respectively, and followed the kinetics of nodule formation. In both cases, no effect of the mutation could be detected on the kinetics of nodule formation and the nitrogenase activity of the plants (Fig 4A and 4B). Also the size and morphology of the Δ*nodD1* and Δ*nodD2* nodules were similar as the WT nodules (Fig 4C). In contrast, when the NF-dependent host plant, *A*. *afraspera*, was inoculated with the *nod*D mutants, no nodules were formed (Δ*nodD1*) or a delay in nodule formation and reduction in nodule number and nitrogenase enzyme activity was observed (Δ*nodD2*) (Fig 5A and 5B). In addition, the ORS285 Δ*nod*D2 nodules lack the atypical superficial outgrowth as observed on nodules of *A*. *afraspera* plants inoculated with WT ORS285 (Fig 5C) [21]. These results indicate that in contrast to the interaction with *A*. *afraspera* both NodD1 and NodD2 do not play a role in the symbiotic interaction between *Bradyrhizobium* ORS285 and *A*. *indica* plants.

### Expression of *nod* genes delays nodulation of *A*. *indica* plants

**Fig 6:**
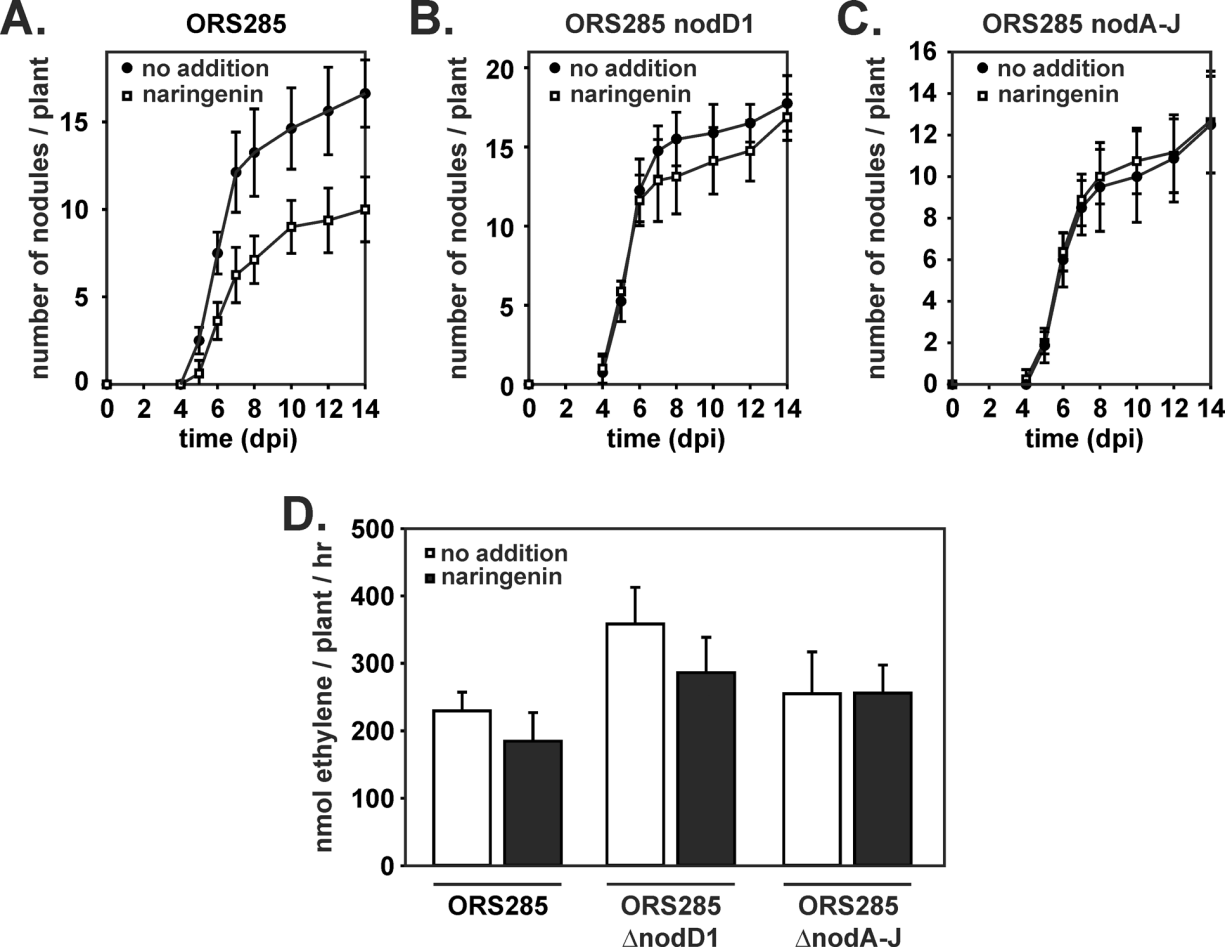
Addition of the *nod*-gene inducing flavonoid naringenin delays nodulation of *A*. *indica* plants (A) Nodulation kinetics of *Bradyrhizobium* ORS285 (B) *Bradyrhizobium* ORS285 Δ*nod*D1 and (C) *Bradyrhizobium* ORS285 Δ*nod*A-J derivatives, on *A*. *indica* plants in the absence and presence of 20 μM of the *nod* gene inducing flavonoid naringenin. The average number of nodules per plant (n = 10) at various days post infection (dpi) is presented. (D) Acetylene reducing activity in *A*. *indica* plants inoculated with *Bradyrhizobium* ORS285 and ORS285 Δ*nod*D1 and Δ*nod*A-J derivatives in the absence and presence of 20 μM naringenin at 15 dpi. The average amount of produced ethylene per hour and per plant is indicated. Error bars represent standard deviations (n = 10).

Above we have shown that NodD1 and NodD2 are not required for the establishment of the symbiosis between ORS285 and *A*. *indica* and that the genes involved in the synthesis of NFs are not induced during this interaction. One can ask the question what will happen when *nod* genes are expressed during the interaction with *A*. *indica*. Does *nod*-gene expression interferes positively or negatively on this interaction or are *A*. *indica* plants completely “blind” to NFs produced upon *nod* gene expression? To address this question, we have inoculated 7-day-old *A*. *indica* seedlings with *Bradyrhizbium* ORS285 in the presence of the *nod*-gene inducing flavonoid naringenin (20 μM). Analysis of the nodulation kinetics showed that the appearance of the first nodules was delayed in the presence of naringenin (Fig 6A). Interestingly, this delay in nodule organogenesis was not observed with the ORS285 Δ*nodD1* and ORS285 Δ*nod*A-J mutant strains suggesting that it is most probably a consequence of NF synthesis (Fig 6B and 6C). Although the addition of naringenin delayed the nodulation of plants by the WT strain (Fig 6A), the nitrogenase activity of the plants at 14 dpi was comparable with that of plants inoculated in the absence of naringenin (Fig 6D). Altogether, these observations suggest that forcing the synthesis of NFs in the ORS285 strain interferes negatively with the establishment of the NF-independent symbiosis but has no effect on the functioning of the nodules.

## Discussion

Previously, we have shown that the symbiotic interaction between photosynthetic *Bradyrhizobium* strain ORS285 and certain tropical aquatic legumes of the *Aeschynomene* genus, e.g. *A*. *afraspera*, involves a classical molecular dialogue (i.e., induction of *nod* genes by host plant flavonoids and subsequent synthesis of LCO)(12). However, with certain species of the *Aeschynomene* genus, e.g. *A*. *indica*, *Bradyrhizobium* ORS285 is able to establish a symbiotic interaction in a NF-independent manner [6]. Little is known about the molecular mechanism of the NF-independent symbiosis of ORS285 with *A*. *indica*. Are NFs synthesized during this interaction? Do NFs interfere positively or negatively with the establishment of the NF-independent symbiosis? Does the regulator NodD known to control the expression of *nod* genes but also many other symbiosis related genes play a role in the NF-independent symbiosis?

Using a *nod*A-*lac*Z reporter strain, we showed that the *nod*-genes are not expressed during the interaction of *Bradyrhizobium* ORS285 with the NF-independent host plant *A*. *indica*. Qualitative analysis of the C18-extracted root exudate of *A*. *afraspera* and *A*. *indica*, respectively, by reversed-phase chromatography showed drastic differences between the two plant species (Fig 1A and 1B). For both root exudates, most compounds isolated after C18 extraction have a typical flavonoid UV-VIS spectrum (S1 and S2 Figs). Moreover, *A*. *indica* root exudate does not inhibit the naringenin-stimulated *nod*-gene expression. The absence of *nod*-gene-inducing flavonoids in *A*. *indica* root exudates is thus likely the explanation for the absence of *nod*A-J gene expression with this plant. The obtained results, however, do not rule out an important role of flavonoids as plant signal and developmental regulator in NF-independent symbiosis. In the NF-independent symbiosis between Frankia and actinorhizal plants, flavonoids have been shown to play an important role [22]. In this context, several pure flavonoids, like kaempherol and quercetein, that do not induce *nod* gene expression stimulate the growth of the ORS285 strain at micromolar concentrations (S3 Fig). A similar growth stimulation is observed with very low amounts of *A*. *indica* root exudate (S3 Fig), suggesting that it contains molecules that are recognized by the ORS285 strain. Currently, we are comparing the transcriptomic profile of ORS285 cells grown in the presence of root exudate from *A*. *afraspera* and *A*. *indica* plants, respectively, to identify genes that are specifically expressed upon interaction with NF-independent *Aeschynomene* species. Also silencing the flavonoid biosynthetic pathway in *A*. *indica* plants will shine a light on the role of flavonoids in NF-independent *Aeschynomene* symbiosis.

NodD proteins are members of the LysR family of transcriptional regulators [23] and play a key role in the activation of transcription of *nod* genes. Like *B*. *diazoefficiens* USDA110, *Bradyrhizobium* ORS285 contains two *nod*D genes, *nod*D1 and *nod*D2. *Nod*D1 or *nod*D2 deletion has no effect on the interaction with the NF-independent *Aeschynomene* species, *A*. *indica*. In contrast, deletion of *nod*D1 completely abolished nodulation of *A*. *afraspera* plants, whereas nodulation of *A*. *afraspera* by the *nod*D2 mutant was retarded.

Nodules of *A*. *afraspera* contain an atypical superficial outgrowth containing bacterial infected cells [21]. Recently, it has been shown that *A*. *afraspera* nodules formed by an ORS285 Type III secretion mutant (*rhcN*) lack this superficial outgrowth [24]. Interestingly, nodules formed by the ORS285 Δ*nod*D2 mutant also lack this outgrowth. In some other rhizobia, NodD1 proteins regulate the expression of *tts*I, which encodes for a regulator of Type III secretion functions [16,25]. Future studies, using *lac*Z/*gus*A reporter strains should clarify if a similar T3SS regulation-cascade is present in the ORS285 strain and if in this cascade NodD2 plays a role.

Here, we demonstrated that *nod*(A-J) genes are not expressed upon contact with *A*. *indica* plants and that deletions of *nod*D1 and *nod*D2, respectively, do not affect the interaction of *Bradyrhizobium* ORS285 with *A*. *indica*. This suggests that in *Bradyrhizobium* ORS285 there is no overlap between the NF-dependent and NF- independent mechanism to interact with different host plants. To investigate what happens when the *nod*-gene-dependent pathway is induced during the interaction with a NF-independent host plant, we have infected *A*. *indica* plants with *Bradyrhizobium* ORS285 in the presence of 20 μM of the *nod* gene- inducing flavonoid naringenin. Naringenin addition to the plant culture medium delays the nodulation of *A*. *indica* plants by *Bradyrhizobium* ORS285 whereas it had no effect on nodulation of *A*. *indica* plants by the Δ*nod*D1 and Δ*nod*A-J mutant strains. This suggests that the observed delay is due to the induction of *nod*-gene expression and consequent NF synthesis. Why would *nod*-gene expression be incompatible with nodulation of NF-independent host plants? One possibility could be that the induction of synthesis of NFs alters the physiology of the ORS285 strain and that this distinct physiology negatively affects the interaction with *A*. *indica* plants. Another possibility is that in NF-independent *Aeschynomene* species (a) dedicated LysM-RLK receptor(s) play a role in the perception of bacterial signals leading to nodule organogenesis. For the classical NF-dependent symbiosis two putative models have been proposed to explain the evolutionary conserved dual function of LysM-RLK receptors in immune response and nodulation. (a) Perception of NFs modulates the balance between different LysM-RLK receptor complexes, favoring a symbiotic complex at the expense of complexes required for immune responses. (b) Early immune responses are co-opted to facilitate symbiont infection. Tight regulation of the receptor complexes at the post-translational level, involving rapid endocytotic turnover, subsequently prevents activation of defense responses (for review, see [26]). Both models can explain how the artificially induced NFs could interfere with the nodulation of NF-independent *Aeschynomene* species when they are recognized and go into competition with the so far unknown bacterial factor(s) inducing nodulation. Currently, we are analyzing genomic/transcriptomic data from the model NF-independent *Aeschynomene* species, *A*. *evenia*, for the presence of LysM-RLK receptors with the goal to analyze their role in the nodule organogenesis signaling cascade. In addition, future experiments using pure NFs and transcriptomic analysis will help to identify how NFs are recognized by NF-independent *Aeschynomene* species and why this interferes with nodule organogenesis.

Here, we showed that the *nod*(A-J) genes of *Bradyrhizobium* ORS285 are only expressed upon contact with the NF-dependent *Aeschynomene* species, *A*. *afraspera*. Although the *nod*D1 containing region is absolutely essential and *nod*D2 stimulatory for the interaction of *Bradyrhizobium* ORS285 with *A*. *afraspera*, no effect of a *nod*D1 or *nod*D2 deletion has been observed for the interaction with the NF-independent *Aeschynomene* species, *A*. *indica*. This suggests that *Bradyrhizbium* ORS285 uses two completely separated mechanisms to interact with NF-dependent and NF-independent host plants. *Bradyrhizobium* ORS285 is thus an ideal model strain to study and compare the two symbiotic mechanisms that photosynthetic bradyrhizobia use to interact with their host plants.

## Supporting Information

S1 Fig UV-VIS spectra of peaks present in the HPLC chromatogram of SepPak C18 extracted root exudate of *A*. *afraspera*. (TIF)

S2 Fig UV-VIS spectra of peaks present in the HPLC chromatogram of SepPak C18 extracted root exudate of *A*. *indica*. (TIF)

S3 Fig *A*. *indica* root exudate and some pure flavonoids stimulate growth of ORS285 cells but do not induce *nod*A gene expression. (A) Absorbance (at 600 nm) and (B) β-galactosidase activity of *Bradyrhizobium* ORS285::*nod*A-*lac*Z cells grown for 24 hrs in the presence of different pure flavonoids (5 μM final concentration) or root exudate (8 μg/ml final concentration) of *A*. *indica* and *A*. *afraspera* plants. The results are from one representative experiment with three technical replicates for each experimental condition.
(TIF)

S1 File Material and methods. (DOCX)

S1 Table Bacterial strains used in this study. (DOCX)

S2 Table Plasmids used in this study. (DOCX)
