# Supplementary figures and images for: NodD1 and NodD2 Are Not Required for the Symbiotic Interaction of Bradyrhizobium ORS285 with Nod-Factor-Independent Aeschynomene Legumes
Source: PLoS One. 2016 Jun 17;11(6):e0157888. doi: 10.1371/journal.pone.0157888 (PMC4912097; doi:10.1371/journal.pone.0157888)

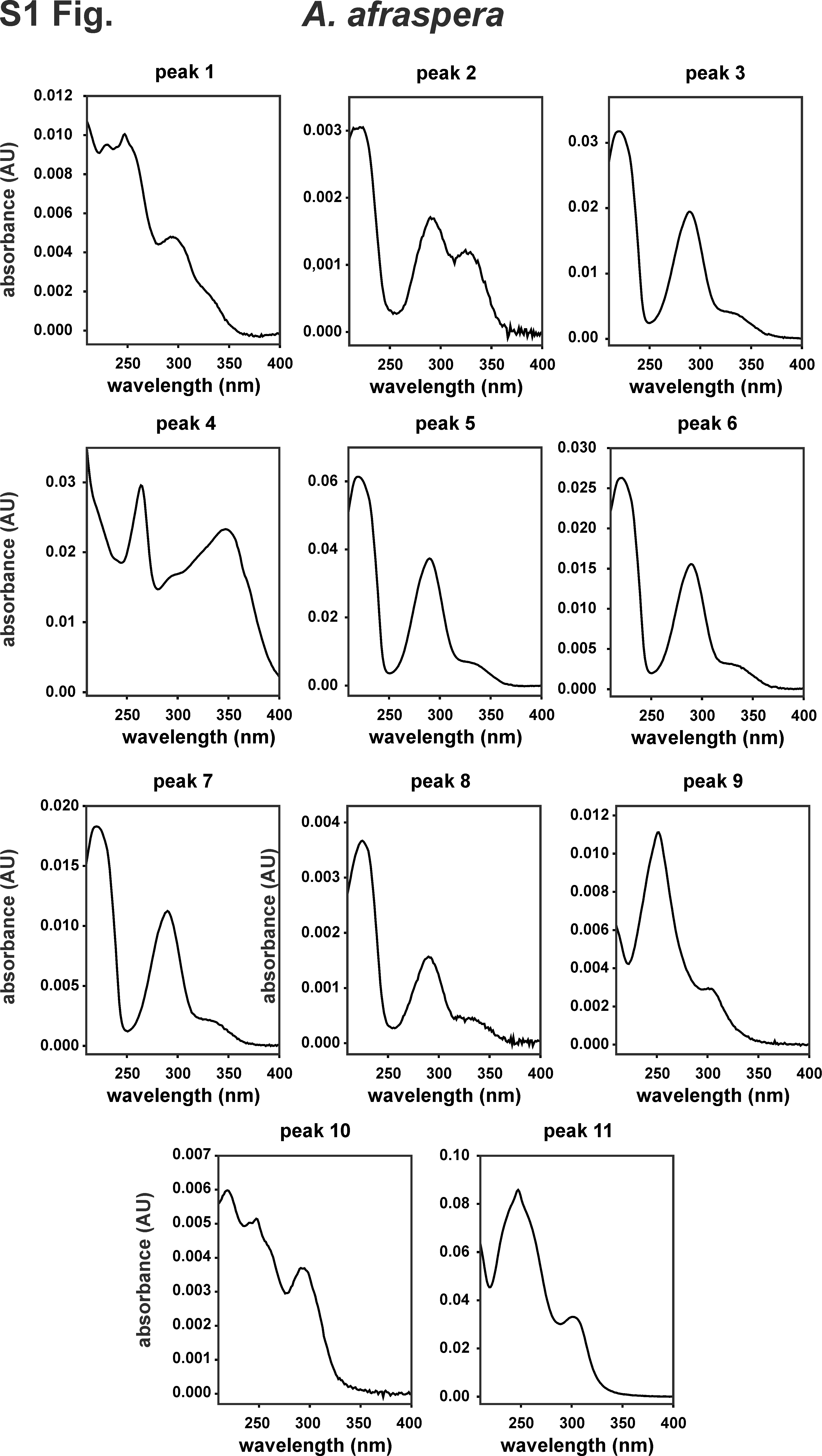

Supplement: S1 Fig — (TIF) [file pone.0157888.s001.tif]

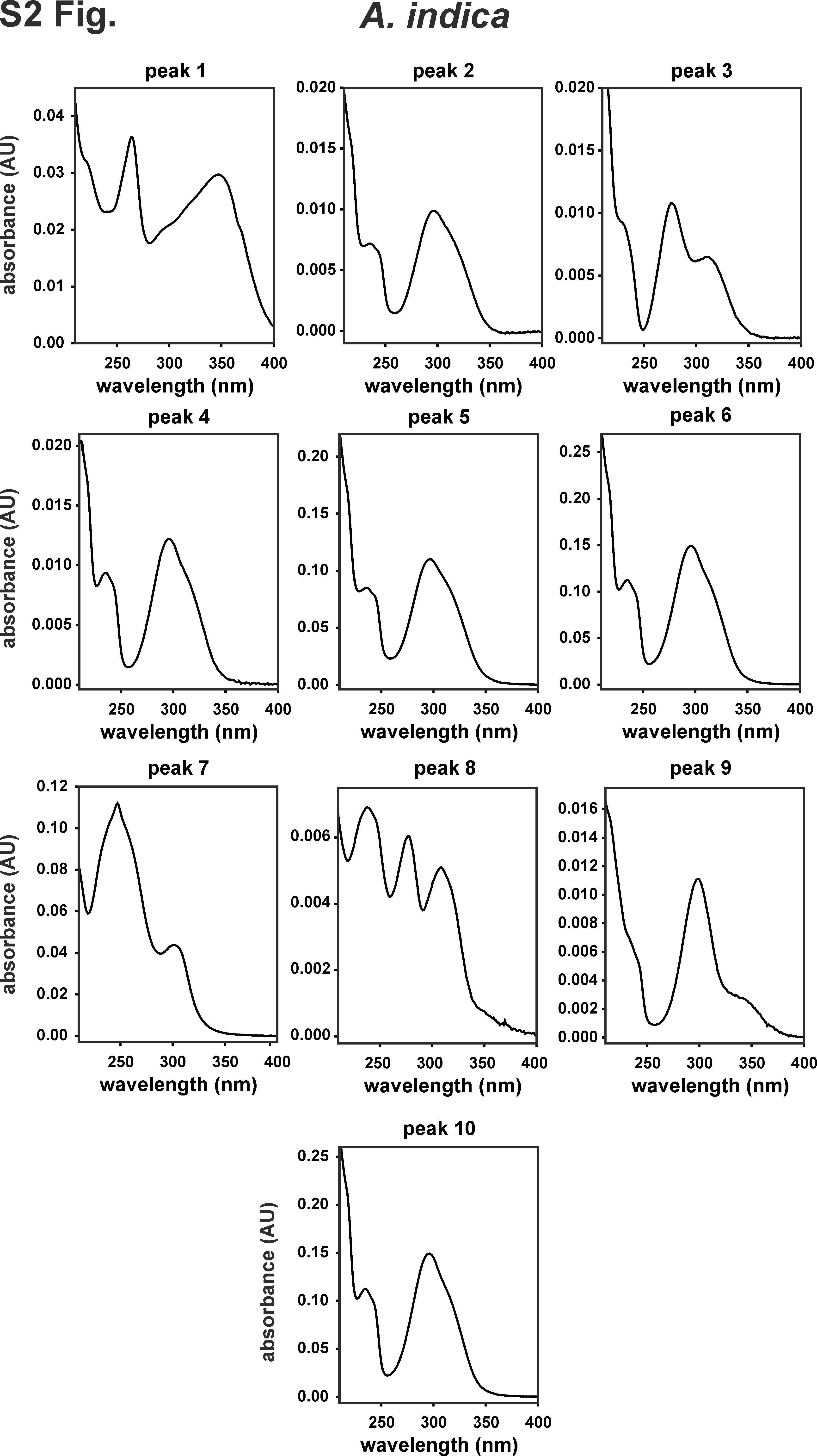

Supplement: S2 Fig — (TIF) [file pone.0157888.s002.tif]

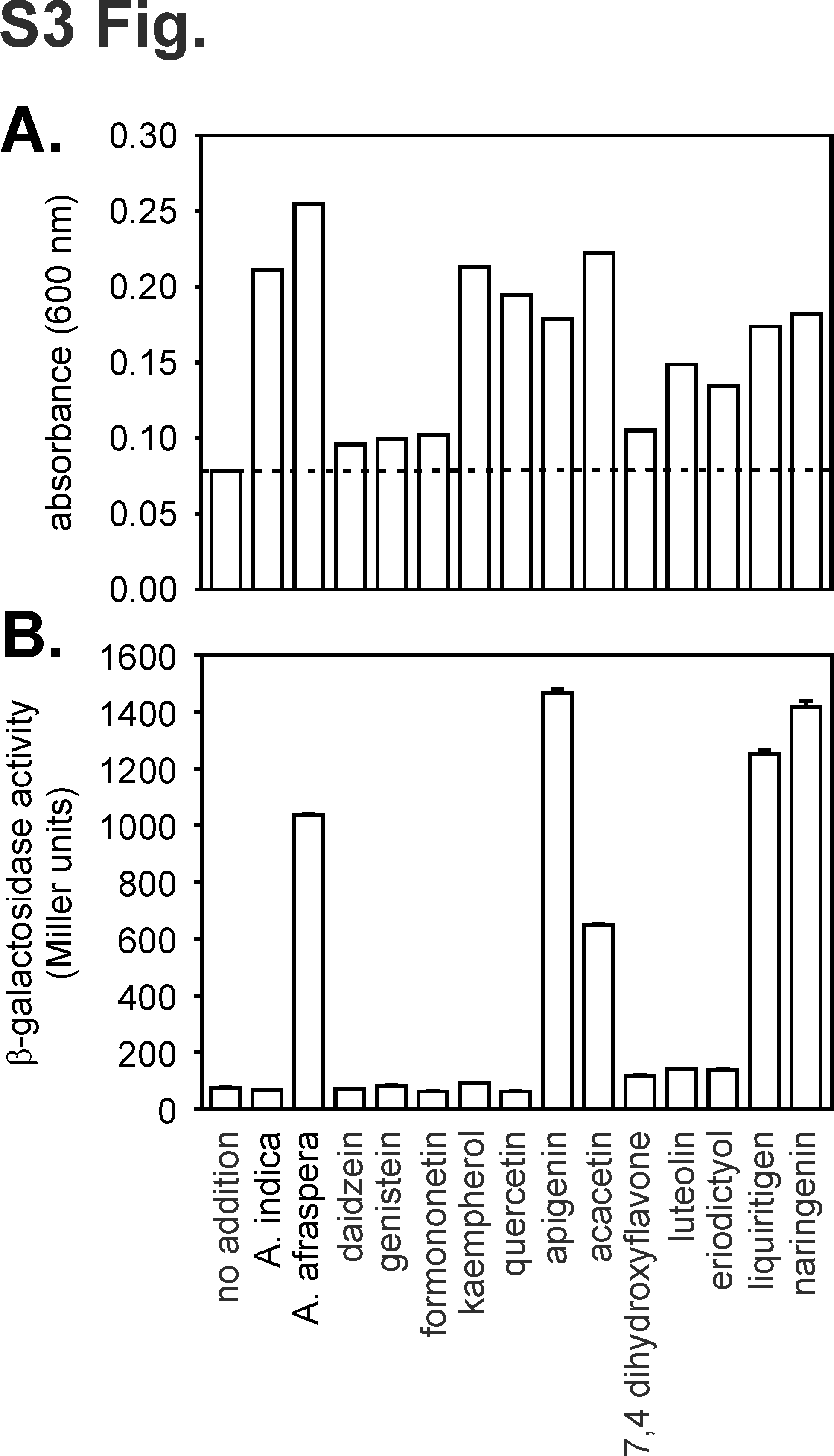

Supplement: S3 Fig — (A) Absorbance (at 600 nm) and (B) β-galactosidase activity of Bradyrhizobium ORS285::nodA-lacZ cells grown for 24 hrs in the presence of different pure flavonoids (5 μM final concentration) or root exudate (8 μg/ml final concentration) of A. indica and A. afraspera plants. The results are from one representative experiment with three technical replicates for each experimental condition. (TIF) [file pone.0157888.s003.tif]
