# Supplementary material for: NodD1 and NodD2 Are Not Required for the Symbiotic Interaction of Bradyrhizobium ORS285 with Nod-Factor-Independent Aeschynomene Legumes
Source: PLoS One. 2016 Jun 17;11(6):e0157888. doi: 10.1371/journal.pone.0157888 (PMC4912097; doi:10.1371/journal.pone.0157888)
Supplement: S1 File — (DOCX) [file pone.0157888.s004.docx]

**S1 File. Material and methods**

To construct *Bradyrhizobium* ORS285 *nod*A-*lac*Z reporter strains, the *lac*Z gene including its ribosome binding site was excised from plasmid pHRP308 [1] using the restriction enzymes *Bam*HI and *Hind*III and inserted into plasmid pJG194-P4694-*mia*A [2] digested with *Bam*HI and *Hind*III. Correct clones were verified by restriction enzyme analysis and the resulting plasmid was named pJG194-*lac*Z. The *nod*A promoter region was amplified by PCR using primers nodA_ORS285_for: 5’- GGCATGCGCAGGTACTACAACGAAGAACG-3’ and nodA_ORS285_rev: 5’- GGATCCATCTAAGCTGCGACCGCATCTACAACC-3’. The resulting DNA fragment was cloned into pGEM-T Easy and transformed into thermos-competent *E.coli* XL2-Blue cells. Correct clones were verified by sequence analysis. For a transcriptional *lac*Z fusion, the *nod*A promoter region was excised with *Sph*I and *Bam*HI and ligated into the suicide vector pJG194-*lac*Z digested with *Sph*I and *Bam*HI. The ligation mix was transformed into *E.coli* XL2 Blue cells and correct clones were selected via DNA restriction enzyme analysis. Plasmids were transformed into CaCl_2_ competent *E.coli* S17.1 cells and mobilized into *Bradyrhizobium* ORS285 using the biparental mating protocol as previously described [2]. To construct a deletion of the *nodA-J* operon of *Bradyrhizobium* ORS285 by crossover PCR the following sets of primers were used. nodA_for: 5’ GCGATCGAGGCGGCGGCGCGAAG 3’; nodA_B-rev: 5’ CCTTTACATCGTGAGACGGATCCATCTAAGCTGCGACCGCATCTAC 3’; nodJ_B-for 5’ GTCGCAGCTTAGATGGATCCGTCTCACGATGTAAAGGAGGAATTCCGTTCAG 3’; nodJ_rev: 5’ CCTCTCTAGGGGCCAGAACGAAC 3’. The resulting DNA fragment was cloned into pGEM-T Easy and transformed into thermocompetent *E.coli* XL2-Blue cells. Correct clones were verified by sequence analysis. For *nod*A-J deletion, the Δ*nod*A-J fragment in pGEM-T Easy was excised with *Apa*I - *Spe*I and ligated into the suicide vector pNPTS139 [5] digested with *Apa*I – *Spe*I. The ligation mixture was transformed into *E.coli* XL2 Blue cells and correct clones were selected via kanamycin resistance (50 µg/ml) and subsequent DNA restriction enzyme analysis. Plasmid pNPT139-Δ*nod*A-J, was transformed into CaCl_2_ competent *E.coli* S17.1 cells. Conjugation, and selection of clones in which the *nod*A-J region was deleted was performed as described above.

To construct a deletion of the *nod*D1 *and nod*D2 gene of *Bradyrhizobium* ORS285 by crossover PCR the following sets of primers were used. *Nod*D1: nodD1_F_A: 5’- GTGTGACGCGACCATATTGCGTTCG-3’; nodD1_R_A:

5’-GCTTAAGCTTGGGGCGCGGATCCTTGACTCGCAGTGAAATTACTCATCG-3’and nodD1_F_B: 5’-GTAATTTCACTGCGAGTCAAGGATCCGCGCCCCAAGCTTAAGCCGACGACG-3’; nodD1_R_B: 5’- GCTTCATTCCGTTCAGCGCGGAACAGACG-3’. *Nod*D2: nodD2_F_A: 5’- GGATCGCTCTAGTCTGCGTCTATAGG-3’; nodD2_R_A: 5’-GCCCTACGATAATGACCTGGATCCTAGATCAAGCCCCTTGAAACGCATATCG-3’ and nodD2_F_B: 5’-CAAGGGGCTTGATCTAGGATCCAGGTCATTATCGTAGGGCGGAGGCCGTTACG-3’; nodD2_R_B: 5’-CGAGAAGCCCCACGAATTGCCGTTGC-3’. The resulting DNA fragment was cloned into pGEM-T Easy and transformed into thermocompetent *E.coli* XL2-Blue cells. Correct clones were verified by sequence analysis. For selection purposes, the chloramphenicol and streptomycin omega interposons from pHP45-Cm [3] and pHRP315 [1], respectively, were isolated as a *Bam*HI fragment and ligated into pGEM-T-Δ*nod*D1 and pGEM-T-Δ*nod*D2, respectively, digested with *Bam*HI. For *nod*D1 deletion, the *nod*D1-Cm^R^ fragment in pGEM-T Easy was excised with *Nco*I-*Spe*I and ligated into the suicide vector pNPTS129 [4]. For *nod*D2 deletion, the *nod*D2-Sm^R^ fragment in pGEM-T Easy was excised with *Apa*I - *Spe*I and ligated into the suicide vector pNPTS139 [5] digested with *Apa*I – *Spe*I. The ligation mixtures were transformed into *E.coli* JM109 cells and correct clones were selected via chloroamphenicol resistance (50 µg/ml; pNPTS129-Δ*nod*D1) or streptomycin resistance (20 µg/ml; pNPTS139-ΔnodD2) and subsequent DNA restriction enzyme analysis. For mobilization into *Bradyrhizobium* ORS285, plasmids pNPTS129-Δ*nod*D1-Cm^R^ and pNPT139-Δ*nod*D2-Sm^R^, which contains a counter selectable *sac*B marker, were transformed into CaCl_2_ competent *E.coli* S17.1 cells. Conjugation, and selection of clones in which the *nod*D1 and *nod*D2 were deleted, respectively, was performed as described above. The *nod*D1 and n*od*D2 deletion in kanamycin sensitive and chloroamphenicol/streptomycin resistant colonies, respectively, was confirmed by PCR.

**References supporting information**

1. Parales RE, Harwood CS. Construction and use of a new broad-host-range lacZ transcriptional fusion vector, pHRP309, for Gram^-^ bacteria. Gene 1993;133: 23-30.

2. Podlešáková K, Fardoux J, Patrel D, Bonaldi K, Novák O, Strnad M, et al. Rhizobial synthesized cytokinins contribute to but are not essential for the symbiotic interaction between photosynthetic Bradyrhizobia and *Aeschynomene* legumes. Mol Plant Microbe Interact 2013; 26: 1232-1238.

3. Fellay R, Frey J, Krisch H. Interposon mutagenesis of soil and water bacteria: a family of DNA fragments designed for *in vitro* insertional mutagenesis of Gram-negative bacteria. Gene 1987;52: 147-154.

4. Tsai J-W, Alley MRK. Proteolysis of the McpA chemoreceptor does not require the *Caulobacter* major chemotaxis operon. J Bacteriol 2000;182: 504-507.

5. Fischer B, Rummel G, Aldridge P, Jenal U. The FtsH protease is involved in development, stress response and heat shock control in *Caulobacter crescentus*. Mol Microbiol 2002;44: 461-478.

6. Simon R, Priefer UB, Puhler A. A broad host range mobilization system for *in vivo* genetic-engineering - transposon mutagenesis in gram-negative bacteria. Bio-Technology 1983;1: 784-791.

7. Yanisch-Perron C, Vieira J, Messing J. Improved M13 phage cloning vectors and host strains: nucleotide sequences of the M13mp18 and pUC19 vectors. Gene 1985;33: 103-119.

8. Molouba F, Lorquin J, Willems A, Hoste B, Giraud E, Dreyfus B, et al. Photosynthetic bradyrhizobia from *Aeschynomene* spp. are specific to stem-nodulated species and form a separate 16S ribosomal DNA restriction fragment length polymorphism group. Appl Environ Microbiol 1999;65: 3084-3094.
