# Supplementary material for: NodD1 and NodD2 Are Not Required for the Symbiotic Interaction of Bradyrhizobium ORS285 with Nod-Factor-Independent Aeschynomene Legumes
Source: PLoS One. 2016 Jun 17;11(6):e0157888. doi: 10.1371/journal.pone.0157888 (PMC4912097; doi:10.1371/journal.pone.0157888)
Supplement: S1 Table — (DOCX) [file pone.0157888.s005.docx]

**S1** **Table**. **Bacterial strains used in this study**.

| **Bacterial strain** | **Relevant characteristics** | **Reference or source** |
| --- | --- | --- |
| *E.coli* XL2 Blue | endA1 gyrA96(nal^R^) thi-1 recA1 relA1 lac glnV44 F'[ ::Tn10 proAB^+^ lacI^q^ Δ(lacZ)M15 Amy Cm^R^] hsdR17(rK^-^ mk^+^) | Stratagene |
| *E.coli* S17.1 | pro res^-^ hsdR17 (rK^-^ mk^+^) recA with an integrated RP4-2-Tc::Mu-Km::Tn7 Tp^R^ | [6] |
| *E. coli* JM109 | endA1 glnV44 thi-1 relA1 gyrA96 recA1 mcrB^+^ Δ(lac-proAB) e14- [F' traD36 proAB^+^ lacI^q^ lacZΔM15] hsdR17(rK^-^ mk^+^) | [7] |
| *Bradyrhizobium* ORS285 | Wild-type strain | [8] |
| *Bradyrhizobium* ORS285 Δ*nod*A-J | *Bradyrhizobium* ORS285 containing a deletion of the *nod*A-J operon | This work |
| *Bradyrhizobium* ORS285 Δ*nod*D1 | *Bradyrhizobium* ORS285 containing a deletion of *nod*D1; Cm^R^ | This work |
| *Bradyrhizobium* ORS285 Δ*nod*D2 | *Bradyrhizobium* ORS285 containing a deletion of *nod*D2; Sm^R^ | This work |
| *Bradyrhizobium* ORS285::*nod*A-*lac*Z | *Bradyrhizobium* ORS285 reporter strain containing a transcriptional fusion of *lac*Z with *nod*A; Kn^R^ | This work |
