# Supplementary material for: NodD1 and NodD2 Are Not Required for the Symbiotic Interaction of Bradyrhizobium ORS285 with Nod-Factor-Independent Aeschynomene Legumes
Source: PLoS One. 2016 Jun 17;11(6):e0157888. doi: 10.1371/journal.pone.0157888 (PMC4912097; doi:10.1371/journal.pone.0157888)
Supplement: S2 Table — (DOCX) [file pone.0157888.s006.docx]

**S2 Table.** **Plasmids used in this study.**

| **Plasmid** | **Relevant characteristics** | **Reference or source** |
| --- | --- | --- |
| pGEM-T Easy | Ap^R^ | Promega |
| pJG194-P4694-miaA | Kn^R^ | [2] |
| pHRP308 | Ap^R^ *lac*Z | [1] |
| pJG194-lacZ | pJG194-P4694-miaA in which *mia*A region is replaced by the *lac*Z region of plasmid pHRP308; Kn^R^ *lac*Z | This work |
| pGEM-T Easy P-*nod*A | pGEM-T Easy containing the *nod*A promoter region of *Bradyrhizobium* ORS285; Ap^R^ | This work |
| pJG194-*nod*A-*lac*Z | Transcriptional fusion of the *nod*A promoter region of *Bradyrhizobium* ORS285 with *lac*Z; Kn^R^ | This work |
| pNPTS129 | Kn^R^ *sac*B | [4] |
| pNPTS139 | Kn^R^ *sac*B | [5] |
| pHP45-Cm | Cm^R^ | [3] |
| pHRP315 | Sm^R^ | [1] |
| pGEM-T Easy Δ*nod*A-J | pGEM-T Easy containing Δ*nod*A-J region; Ap^R^ | This work |
| pGEM-T Easy Δ*nod*D1 | pGEM-T Easy containing Δ*nod*D1; Ap^R^ | This work |
| pGEM-T Easy Δ*nod*D1-Cm | pGEM-T Easy containing Δ*nod*D1 region region and omega interposon of pHP45-Cm; Ap^R^ Cm^R^ | This work |
| pGEM-T Easy Δ*nod*D2 | pGEM-T Easy containing Δ*nod*D2; Ap^R^ | This work |
| pGEM-T Easy Δ*nod*D2-Sm | pGEM-T Easy containing Δ*nod*D2 and omega interposon of pHRP315; Ap^R^ Sm^R^ | This work |
| pNPTS139-ΔnodA-J | pNPTS139 containing Δ*nod*A-J region; Kn^R^ *sac*B | This work |
| pNPTS129-Δ*nod*D1-Cm | pNPTS129 containing Δ*nod*D1 region and omega interposon of pHP45-Cm; Kn^R^ Cm^R^ *sac*B | This work |
| pNPTS139-Δ*nod*D2-Sm | pNPTS139 containing Δ*nod*D2 region and omega interposon of pHRP315; Kn^R^ Sm^R^ *sac*B | This work |
